# From suspected brain malignancy to Erdheim-Chester disease: the role of PET imaging and bone marrow biopsy in diagnosis

**DOI:** 10.1093/omcr/omag049

**Published:** 2026-05-10

**Authors:** Abdulrahman Al-Abdulmalek, Stephane Isnard, Andrew A Watters, Jean-Pierre Routy

**Affiliations:** Division of hematology, McGill University Health Centre, 1001 boulevard Decarie, Montreal, QC, H4A3J1, Canada; Research Institute of the McGill University Health Centre, 1001 boulevard Decarie, Montreal, QC, H4A3J1, Canada; Department of Pathology, McGill University, 1001 boulevard Decarie, Montreal, QC, H4A 3J1, Canada; Division of hematology, McGill University Health Centre, 1001 boulevard Decarie, Montreal, QC, H4A3J1, Canada; Research Institute of the McGill University Health Centre, 1001 boulevard Decarie, Montreal, QC, H4A3J1, Canada

**Keywords:** Erdheim-Chester disease (ECD), BRAF V600E mutation, positron emission tomography (PET), bone marrow biopsy, melanoma

## Abstract

Erdheim-Chester Disease (ECD) is an extremely rare non-Langerhans cell histiocytosis involving MAPK pathway mutations, notably BRAFV600E. We report a 60-year-old male with a history of BRAF-negative malignant melanoma and a new melanoma *in situ*, who presented with diabetes insipidus, hypogonadism, ataxia, blurred vision, dysarthria, headache, personality changes, and disturbed sleep patterns with kicking and mumbling. Neurological and systemic features raised suspicion for malignancy. PET imaging was more informative than CT or MRI and identified widespread FDG-avid skeletal and soft tissue lesions. Because of skeletal lesions, a bone marrow biopsy was performed despite a normal complete blood count and proved valuable as compared to biopsy of less accessible tissues. Histopathology from skin and bone marrow confirmed ECD with BRAFV600E mutation. This case highlights the diagnostic role of PET and bone marrow biopsy in ECD diagnosis. Multidisciplinary evaluation and targeted therapy with a BRAF inhibitor led to symptom resolution.

## Introduction

Erdheim-Chester Disease (ECD) is an extremely rare myeloid neoplasm caused by non-Langerhans foamy CD68 + CD1a- histiocyte proliferation and infiltration within various tissues [1, 2]. First described in 1930, ECD typically affects middle-aged adults, with a male predominance, and manifests with a wide range of clinical features that can involve nearly every organ system including long bones, skin, cardiovascular system, kidneys, and central nervous system, often resulting in synchronous multisystem involvement which poses a diagnostic challenge [1–3].

The pathogenesis of ECD involves mutations activating the Mitogen-Activated Protein Kinase (MAPK) pathway, particularly the BRAFV600E mutation, found in approximately 57%–70% of cases [1–4], which has opened avenues for BRAF inhibitors as targeted therapies.

Herein, we present how positron emission tomography (PET), bone marrow and skin biopsies guided the diagnosis of ECD in a 60-year-old male.

## Case report

A 60-year-old male with a history of coronary artery disease in 2023, localized skin malignant melanoma (excised in 2022, BRAF-negative), diabetes insipidus (May 2023), and hypogonadotropic hypogonadism (January 2024), who presented with ataxia, blurred vision, dysarthria and headaches. His family noted personality changes, and kicking and mumbling during his sleep.

He demonstrated subtle dysarthria, full extraocular movements with mild horizontal nystagmus on left lateral gaze, and subtle right-sided facial drop with forehead sparing. Heel-to-shin testing was positive on the right side, and he exhibited a wide-based gait with a positive Romberg sign. Dermatologic examination revealed an isolated red-brown papule on the right leg and a dark skin lesion on his nose.

### Investigations

Laboratory investigations showed normal complete blood count and metabolic panels (Table 1), and infectious and auto-immune testing were negative.

**Table 1:** Laboratory investigations.

Test	Result
Hemoglobin	143 g/l
White Blood Cell Count	9.10 × 10^9/l
Platelet Count	234 × 10^9/l
Abs. Neutrophil	4.8 × 10^9/l
Abs. Lymphocyte	3.2 × 10^9/l
Abs. Monocyte	0.8 × 10^9/l
Abs. Eosinophil	0.3 × 10^9/l
Abs. Basophil	0.07 × 10^9/l
Creatinine	69 μmol/l
Potassium	4.1 mmol/l
Chloride	105 mmol/l
Sodium	141 mmol/l
ALT	16 U/l
ALP	76 U/l
Total Bilirubin	13 μmol/l
C-Reactive Protein	15.9 mg/l

To assess neurological manifestations, a brain magnetic resonance imaging (MRI) was performed and revealed multiple enhancing lesions in the brainstem and bilateral orbital masses encasing the optic nerves. A spine MRI demonstrated abnormal neuroforaminal and prevertebral infiltration with enhancement at the thoracic and lumbar regions, as well as the presacral area. These MRI findings raised suspicion for neurosarcoidosis or primary or secondary brain malignancy.

Cerebrospinal fluid analysis yielded negative for malignant cells or infection. Due to concern for metastatic disease, PET was performed and revealed multiple FDG-avid lesions, including bilateral orbital, extensive soft tissue infiltration around the thoracic aorta, coronary arteries, pericardial masses, and long bones in the lower extremities, suggestive of ECD. The PET also showed retroperitoneal involvement with encasement of the kidneys, also characteristic of ECD (Fig. 1).

**Figure 1:**
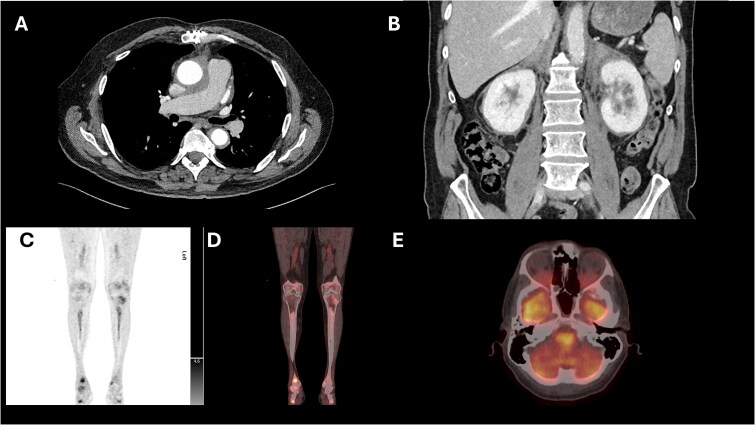
Patient imaging results. CT findings of periaortic soft tissue thickening (A) and perinephric soft tissue infiltration encasing the kidneys and extending into the renal hilum, creating a ‘hairy kidney’ appearance (B). PET scan of the lower limbs showed increased FDG uptake in both distal femur and proximal and distal tibiae as well as both feet corresponding to sclerosis (C and D). FDG uptake within soft tissue densities projected around both optic nerves (E).

As FDG-avid skeletal lesions were observed by PET, bone marrow biopsy and aspiration were performed.

### Other radiographic findings

Abdominal CT showed perinephric soft tissue infiltration encasing the kidneys and extending into the renal hilum, creating a ‘hairy kidney’ appearance, characteristic of ECD. The adrenal glands were enlarged with periadrenal soft tissue densities and splenomegaly. Retroperitoneal and mesenteric soft tissue infiltration were associated with loss of pancreatic lobulations.

### Histopathology

The leg papule skin biopsy showed a histiocytic dermal infiltrate with chronic inflammation, consistent with histiocytosis. Immunohistochemistry was positive for CD68, CD163, and S100 protein, negative for CD1a and Langerin. Strong BRAFV600E positivity supported the ECD diagnosis (Fig. 2). The biopsy of the nose lesion revealed melanoma in situ with focal early dermal invasion and was negative for BRAFV600E.

**Figure 2:**
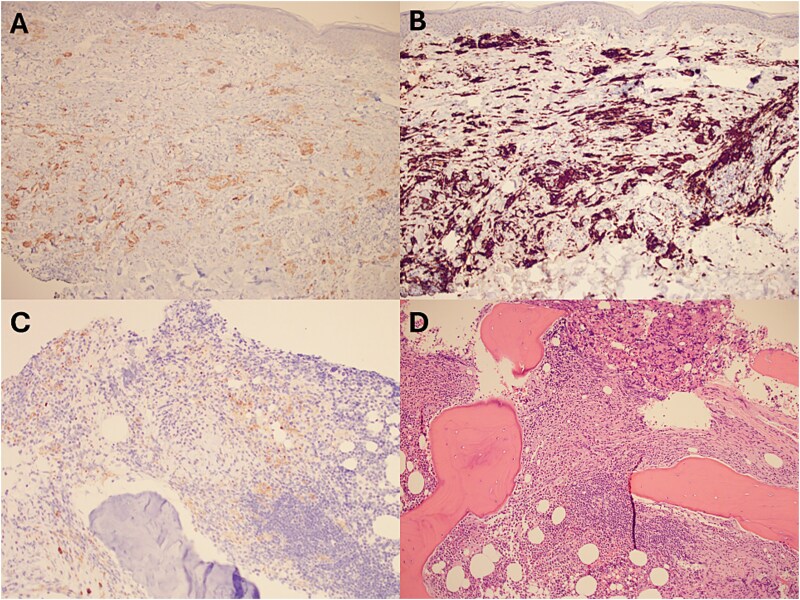
Histopathology assessment. (A) Skin H&E stain with a diffuse lymphohistiocytic dermal infiltrate (10×). (B) Strong BRAFV600E staining of the dermal histiocytic cells (10×). (C) Bone marrow H&E stain with a focal histiocytic infiltrate (10×). (D) Bone marrow staining shows histiocytes positive for BRAFV600E.

A bone marrow biopsy of the left iliac crest showed a normal myeloid to erythroid ratio and an adequate number of megakaryocytes. Increased reticulin and collagen staining, along with aggregates of foamy and mononuclear histiocytes, were observed, consistent with ECD. Immunohistochemical staining was positive for the expression of CD45, CD3, CD20, CD163, CD68, CD34 and CD117 cell markers. Myeloperoxidase was positive in myeloid elements, while CD71 was positive in erythroid elements. S100, CD1a, and ALK were negative. BRAFV600E mutation was detected (Fig. 2).

### Treatment and outcome and follow-up

The patient was initiated on the BRAF inhibitor Vemurafenib 480 mg orally twice daily [1]. Withing two weeks, he experienced clinical improvement with resolution of dizziness, headaches, visual symptoms, and improved control of diabetes insipidus.

At the 4-week follow-up, blood liver enzymes were elevated, despite normal values prior to treatment (Table 2). Due to known interaction with statins, Vemurafenib dose was reduced to 240 mg orally twice daily and rosuvastatin (Crestor®) was held [5]. A repeated liver function test 2 weeks later showed improvement. Four weeks later, liver enzymes had returned to baseline (Table 2).

**Table 2:** Liver profile before and throughout Vemurafenib treatment.

	Pre-treatment	4 weeks post treatment	2 weeks post dose reduction/crestor cessation	6 weeks post dose reduction
AST (U/l)	21	55	22	30
ALT (U/l)	33	107	66	45
GGT (U/l)	not done	481	104	26
ALP (U/l)	76	240	not done	71
Total bilirubin (μmol/l)	12	23	not done	12

AST = aspartate aminotransferase, ALT = alanine aminotransferase, GGT = Gamma-Glutamyl Transferase, ALP = alkaline phosphatase.

Three months after initiating Vemurafenib, a PET scan showed stable, minimally hypermetabolic infiltration of mesenteric and retroperitoneal fat. Bone metabolism appeared normal and retro-orbital lesions were no longer detectable. A brain MRI showed reduction in bilateral retro-orbital masses, pituitary stalk lesion and brainstem involvement.

## Discussion

This case exemplifies the diagnostic challenge associated with the multisystem presentation of ECD. Neurological dysfunction and MRI led to a suspected brain malignancy or neurosarcoidosis [6, 7]. Performed due to malignancy suspicion, PET demonstrated perivascular, retroperitoneal and long bone involvement, shifting the diagnosis toward ECD [1, 8].

In addition to a skin lesion biopsy, bone marrow biopsy and aspiration allowed minimally invasive assessment of FDG-avid region and showed histiocytic infiltration despite normal CBC as well as presence of BRAFV600E mutation. In a study, seven out of eight ECD cases showed bone marrow involvement with three of them having normal CBC [1]. Current recommendations recommend bone marrow assessment upon unexplained cytopenia or monocytosis [9]. Our case indicates that PET guided bone marrow biopsy despite normal CBC was instrumental in ECD diagnostic.

This case highlights the occurrence of ECD in a patient with a history of skin melanoma and a newly diagnosed melanoma. Although myeloid neoplasms have been linked to ECD, associations with other malignancies like melanoma are rare in approximatively 10% of patients [2]. In our patient, both the previous and newly diagnosed melanoma were BRAFV600E-negative. Functional evidences show that ECD may originate from hematopoietic progenitor cells rather than tissue-resident macrophages, suggesting that some cases may be linked to clonal hematopoiesis. Our case indicates that distinct neoplastic mutations can coexist rather than sharing a single oncogenic pathway.

Overall, our case indicates that PET outperformed MRI/CT-scan and guided bone marrow biopsy despite normal CBC to assess hypermetabolic regions, leading to ECD diagnostic.

## Consent

The patient provided informed written consent for the preparation of this case report and publication of data and pathological image in an open access journal.

## Guarantor

Dr Jean-Pierre Routy is the guarantor of this case.

## References

[1] Goyal G, Heaney ML, Collin M. et al. Erdheim-Chester disease: consensus recommendations for evaluation, diagnosis, and treatment in the molecular era. Blood. 2020;135:1929–45. 10.1182/blood.2019003507 32187362

[2] Abdelfattah AM, Arnaout K, Tabbara IA. Erdheim-Chester disease: a comprehensive review. Anticancer Res 2014;34:3257–61. 24982329

[3] Estrada-Veras JI, O’Brien KJ, Boyd LC. et al. The clinical spectrum of Erdheim-Chester disease: an observational cohort study. Blood Advances 2017;1:357–66. 10.1182/bloodadvances.2016001784 28553668 PMC5446206

[4] Badalian-Very G, Vergilio JA, Degar BA. et al. Recurrent BRAF mutations in Langerhans cell histiocytosis. Blood. 2010;116:1919–23. 10.1182/blood-2010-04-279083 20519626 PMC3173987

[5] Piscitelli J, Reddy MB, Wollenberg L. et al. Clinical evaluation of the effect of Encorafenib on bupropion, Rosuvastatin, and Coproporphyrin I and considerations for statin Coadministration. Clin Pharmacokinet 2024;63:483–96. 10.1007/s40262-024-01352-9 38424308 PMC11052825

[6] Zahergivar A, Firouzabadi FD, Homayounieh F. et al. Central nervous system involvement in Erdheim-Chester disease: a magnetic resonance imaging study. Clin Imaging 2024;115:110281. 10.1016/j.clinimag.2024.110281 39270429

[7] Boyd LC, O’Brien KJ, Ozkaya N. et al. Neurological manifestations of Erdheim–Chester disease. Ann Clin Transl Neurol 2020;7:497–506. 10.1002/acn3.51014 32227455 PMC7187721

[8] García-Gómez FJ, Acevedo-Báñez I, Martínez-Castillo R. et al. The role of 18FDG, 18FDOPA PET/CT and 99mTc bone scintigraphy imaging in Erdheim–Chester disease. Eur J Radiol 2015;84:1586–92. 10.1016/j.ejrad.2015.04.022 25975897

[9] Tzankov A, Kremer M, Leguit R. et al. Histiocytic cell neoplasms involving the bone marrow: summary of the workshop cases submitted to the 18th meeting of the European Association for Haematopathology (EAHP) organized by the European bone marrow working group, Basel 2016. Ann Hematol 2018;97:2117–28. 10.1007/s00277-018-3436-0 30084011 PMC6182749

